# Recent Advances in Models of Immune-Mediated Drug-Induced Liver Injury

**DOI:** 10.3389/ftox.2021.605392

**Published:** 2021-04-27

**Authors:** Farah Tasnim, Xiaozhong Huang, Christopher Zhe Wei Lee, Florent Ginhoux, Hanry Yu

**Affiliations:** ^1^Innovations in Food & Chemical Safety Programme, A*STAR, Singapore, Singapore; ^2^Institute of Bioengineering and Nanotechnology, The Nanos, Singapore, Singapore; ^3^Department of Physiology, Yong Loo Lin School of Medicine, National University of Singapore, Singapore, Singapore; ^4^Singapore Immunology Network, Singapore, Singapore; ^5^School of Biological Sciences, Nanyang Technological University, Singapore, Singapore; ^6^Shanghai Institute of Immunology, Shanghai JiaoTong University School of Medicine, Shanghai, China; ^7^Translational Immunology Institute, SingHealth Duke-NUS Academic Medical Centre, Singapore, Singapore; ^8^National University of Singapore (NUS) Graduate School for Integrative Sciences and Engineering, Centre for Life Sciences, Singapore, Singapore; ^9^T-Labs, Mechanobiology Institute, Singapore, Singapore; ^10^Critical Analytics for Manufacturing Personalised-Medicine Interdisciplinary Research Groups (CAMP-IRG), Singapore-Massachusetts Institute of Technology Alliance for Research and Technology, Singapore, Singapore

**Keywords:** immune-mediated liver injury, co-culture, human iPSC, macrophages, Kupffer cells, hepatocytes, inflammation

## Abstract

Hepatic inflammation is a key feature of a variety of liver diseases including drug-induced liver injury (DILI), orchestrated by the innate immune response (Kupffer cells, monocytes, neutrophils, dendritic cells) and the adaptive immune system (T cells and natural killer T cells). In contrast to acute DILI, prediction of immune-mediated DILI (im-DILI) has been more challenging due to complex disease pathogenesis, lack of reliable models and limited knowledge of underlying mechanisms. This review summarizes *in vivo* and *in vitro* systems that have been used to model im-DILI. In particular, the review focuses on state-of-the-art *in vitro* human-based multicellular models which have been developed to supplement the use of *in vivo* models due to interspecies variation and increasing ethical concerns regarding animal use. Advantages of the co-cultures in maintaining hepatocyte functions and importantly, introducing heterotypic cell-cell interactions to mimic inflammatory hepatic microenvironment are discussed. Challenges regarding cell source and incorporation of different cells with physical cell-cell contact are outlined and potential solutions are proposed. It is likely that better understanding of the interplay of immune cells in liver models will allow for the development of more accurate systems to better predict hepatotoxicity and stratification of drugs that can cause immune-mediated effects.

## Introduction

Drug-induced liver injury (DILI) is a major cause of patient morbidity and mortality and remains a serious problem for clinicians, pharmaceutical industries and regulatory bodies (Hoofnagle and Björnsson, 2019). Although the complex mechanisms of DILI are not fully understood, both non-immune-mediated and immune-activated mechanisms have been proposed (Waddington et al., 2018). In contrast to non-immune-mediated DILI, prediction of immune-mediated DILI (im-DILI) has been more challenging due to intricate disease pathogenesis, limited knowledge of underlying mechanisms and lack of reliable models (Lee and Senior, 2005). The complexity of the system arises in part due to the liver being a unique immunological organ which harbors a rich, diverse array of complementary and interacting innate and adaptive immune cells (Adams et al., 2010; Waddington et al., 2018).

The resident innate immune cells in the liver include macrophages, called Kupffer cells (KCs), dendritic cells (DCs), neutrophils, natural killer (NK) cells and natural killer T (NKT) cells (Liaskou et al., 2012), while CD4^+^ and CD8^+^ T cells comprise the adaptative immune system (Waddington et al., 2018). In addition to these cells, the unique vascularization of the liver also allows for the recruitment of circulating leukocytes upon activation of relevant signaling pathways (Liaskou et al., 2012; Mak and Uetrecht, 2017). These immune cells are critical for establishing the balance between host defense against pathogens and tolerance to self- and harmless antigens. In the absence of external stimulus, this balance ensures optimal liver homeostasis. However, in the presence of an infection and/or inflammation, or upon exposure to certain compounds, the balance can be dysregulated, leading to immune cell activation (Krenkel et al., 2014; Waddington et al., 2018). Evolving studies suggest that the direct effects of drugs on hepatic cells might itself be an initiating factor to trigger such an immune response, as exemplified by the case of Acetaminophen (APAP)-mediated immune response (Krenkel et al., 2014). Briefly, KCs form the first line of defense and recognize invading pathogens or necrotic cells resulting from drug injury such as APAP overdose via danger-associated-molecular patterns (DAMPs) and pathogen-associated molecular patterns (PAMPs). KCs release cytokines [e.g., interleukin-6 (IL-6), tumor necrosis factor α (TNFα)] and chemokines (e.g., C-X-C motif ligand (CXCL)-1/3/8) upon activation, leading to acute inflammation marked by increased acute phase proteins such as C-reactive protein and complement components (Liaskou et al., 2012). In parallel, released cytokines lead to the upregulation of adhesion molecules in hepatic sinusoidal endothelial cells, while chemokines attract neutrophils and monocytes to the liver. Monocytes differentiate into tissue macrophages (MoMϕ), and together with the originally resident macrophages (KCs) further release cytokines and factors that promote the survival of neutrophils and hence maintain inflammation. In addition, KC-activated (Krenkel et al., 2014) and DC-activated T-cells (Ogese et al., 2017) have been proposed to be important mediators of inflammation, although exact mechanisms have not been fully clarified yet.

Moreover, the contribution of immune cells is not only restricted to amplification of inflammation following the initial onset of immune activation. Roles of KCs, MoMϕ and neutrophils have been reported in tissue repair and alleviation of injury (Shan and Ju, 2019). Specifically, KC and neutrophil death have been shown to promote tissue repair by switching MoMϕ from pro- to anti-inflammatory states (Triantafyllou et al., 2018; Yang et al., 2019). These MoMϕ in turn induce apoptosis of neutrophils, further resolving inflammation (Holt et al., 2008). Overall, immune cells have a critical role to play in both the augmentation and reduction of inflammation, and by extension in the progression and severity of im-DILI. Therefore, the key to predicting and preventing im-DILI is to first understand the complex interactions between immune cells and other parenchymal/non-parenchymal cells in the liver.

*In vivo* animal models have been used to model im-DILI (Shenton et al., 2004; Ng et al., 2012; Kuna et al., 2018; Uetrecht, 2019); however, treatment of animals with drugs that are known to cause im-DILI in humans do not cause consistent liver injury in animals, even at high doses (Uetrecht, 2019). Even when liver injury is observed, the characteristics are different from that of humans and possibly manifest through different mechanisms (Ng et al., 2012). In addition to the bottleneck of interspecies variation, animal models are expensive and there is increasing pressure toward reducing animal use in research. Owing to these challenges, significant efforts have been put into developing *in vitro* models to not necessarily replace, but rather to supplement *in vivo* animal models. Here, we describe important *in vivo* and *in vitro* systems that have been used to model im-DILI, with emphasis on the recent advances in multi-cellular *in vitro* systems comprising of immune cells to better recapitulate the complexity of the *in vivo* conditions, aiming to improve the current predictivity of *in vitro* liver models.

## Animal Models

In trying to understand DILI, animal models (especially mice and rat models) have long occupied center stage as the gold standard, becoming part of the regulatory requirement for drug development in almost all jurisdictions. The data gathered using these models has been invaluable, affording us a systemic view of the effects of drug absorption and metabolism, as it was through studying these systems that the role of the immune system in DILI came into the spotlight. Apart from the evident connection between hepatocyte death and the inflammatory response brought on by the DAMPs released during the apoptosis or necrosis of these cells (Martin-Murphy et al., 2010), there are also much more nuanced associations between DILI and the immune system that have been uncovered with the aid of depletion or knockout models.

For instance, KCs, the resident macrophages of the liver and the largest immune population as well, have been implicated in the progression of im-DILI in murine models. However, it is still unclear whether they play a protective or aggravating role. Mice that have been treated with Gadolinium Chloride (GdCl3) to inhibit KC activity prior to administration of APAP, appeared to be more resistant to injury, with lower levels of ALT observed after 8 h as compared to control mice (Michael et al., 1999). Paradoxically, using a chlodronate-liposome based depletion approach seemed to have the opposing effect, as complete depletion of the KCs resulted in increased ALT serum levels, indicative of liver injury (Ju et al., 2002). However, these contrasting results might just be highlighting different aspects of macrophage biology, as GdCl3 does not deplete macrophages, but rather suppresses their activation and reactive oxygen species (ROS) production. The cross-talk between gut microbes and KCs further complicates the relationship between KCs and im-DILI. Lipopolysaccharide (LPS) has been identified as a possible contributing factor to liver injury, as it was observed that mice that had been treated with GdCl3 were more resistant to alcohol-induced liver injury, similar to mice that have had their gut microbes depleted by antibiotics (Thurman et al., 1998). This reduction in liver injury was also seen in animals treated with anti-TNFα antibodies, hinting at the possible relationship between KC activation via LPS-induced TNFα and DILI severity. Later studies strengthened this connection, as mice that were given the broad spectrum antibiotic Trovafloxacin (TVX) alone did not develop DILI, while mice that received both LPS and TVX did (Shaw et al., 2007), similarly in a TNFα-dependent manner. KCs have also been shown to directly modulate the metabolic activity of hepatocytes (Odegaard et al., 2008), although the extent to which this contribute to DILI is still unknown and warrants further exploration. Equally intriguing is the possibility of macrophages participating directly in the metabolism of drugs, as several species of cytochrome P450 (CYP450) have been found to be expressed by macrophages in both rodents (Koop et al., 1991) and humans (Hodges et al., 2001).

The use of animal models has also revealed some role for NKT cells, and to a lesser extent NK cells, in the development of im-DILI. An initial study reported that NKT and NK cells contributed to liver injury in the case of APAP-induced toxicity in mice, and that depletion of these populations could significantly reduce the damages seen in these animals (Liu et al., 2004). This was later shown to be an artifact caused by the use of DMSO as the solvent for the APAP (Masson et al., 2008), which triggered the recruitment and activation of NKT and NK cells in the liver. In the absence of DMSO, NKT cells appeared to have a protective effect against APAP induced liver injury instead, with both CD1d^−/−^ and Jα18^−/−^ mice having elevated ALT serum levels as compared to the WT control (Martin-Murphy et al., 2013). However, this does not seem to be the case for all drugs though, as CD1d^−/−^ mice were more resistant to the volatile anesthetic, halothane-induced liver damage, with a corresponding reduction in neutrophil infiltration as well (Cheng et al., 2010).

Although animal models have been immensely helpful, these contradictory results highlight the need for alternative models that can allow us to study specific cell-cell interactions with adjustable complexities to complement these *in vivo* systems.

## *In vitro* Models

### Soluble Factor-Driven Single Cultures in Combination With Inflammatory Mediators

**Figure 1:**
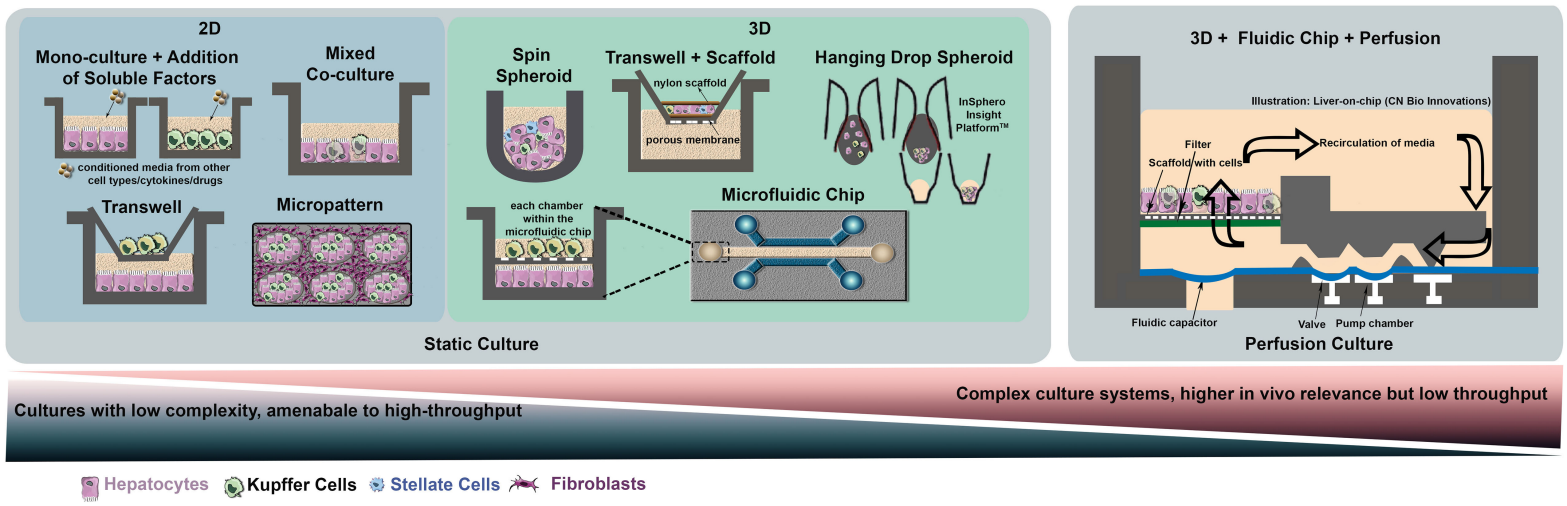
Selection of simple and complex models used for *in-vitro* liver co-cultures. On the left-hand side, various platforms for static cultures are presented, ranging from simple co-cultures in multi-well plates to more complex systems such as microtissue (hanging drop method), micropatterned surfaces, 3D spheroids formed using scaffolds or microfluidic chips. On the right-hand panel, a macroscopic 3D culture on a chip incorporating a perfusion system is represented using the bioreactor developed by CN Bio Innovations.

Possibly with the aim to minimize complexity of the models, some studies have tried to mimic inflammatory effects by treating liver parenchymal cells (hepatocytes) with im-DILI-associated cytokines or conditioned media from immune cells instead of directly adding immune cells into the culture (Cosgrove et al., 2009, 2010; Melino et al., 2012; Saab, 2013) (Table 1, Figure 1). It has been demonstrated that conditioned media of THP-1 macrophages caused morphological and functional changes in hepatocytes (Melino et al., 2012). HepG2 cell line deployed in a high-throughput system with a combination of LPS/cytokine/drug co-treatment demonstrated inflammation-associated hepatotoxic potential with four im-DILI drugs (Saab, 2013). Primary rat hepatocytes (pRHs), human hepatocytes (pHHs) and HepG2 utilized in a high throughput drug–cytokine co-treatment approach identified drug-cytokine hepatotoxicity synergies for multiple im-DILI-associated hepatotoxicants, largely potentiated by TNFα, interleukin-1α (IL-1α), and LPS (Cosgrove et al., 2009). A follow up study using this approach in combination with multiplex phosphoprotein signaling demonstrated the roles of different protein kinase-mediated signaling pathways in drug/cytokine synergy and hepatotoxicity (Cosgrove et al., 2010). These studies may be valuable for elucidating mechanisms in human cells due to their compatibility with high throughput, multiplexed measurements; however, the lack of direct immune cell interaction and crosstalk with hepatocytes remain unaddressed.

**Table 1:** *In vitro* models comprising of soluble factors from conditioned media of hepatocytes and immune cells.

**References**	**Cells**	**2D/3D**	**Duration**	**Stimuli (μg/ml)**	**im-DILI Compounds**	**Findings**	**Disadvantages**
Melino et al. (2012)	HepG2, Huh7, THP-1	CM	24 h	NA	NA	THP1 induce inflammatory responses in HepG2	No drug testing data.
Saab (2013)	HepG2	CM	24h	LPS (20), TNFα (0.1)	Chlorpromazine, TVX, Ranitidine, Sulindac, DIC, TGZ	Drug+inflammation → MRP2 → cholestasis	Lack of immune cells.
Cosgrove et al. (2009, 2010)	pHHs, HepG2, pRHs	SC/monolayer	12/24/48 h	LPS (10), TNFα (0.1), IFN (0.1), IL1α (0.02)	TVX, Ranitidine, nefazodone, nimesulide, telithromycin +90	19% of hepatotoxicants showed cytokine synergy effects within 100*Cmax; follow-up study unveiled the mechanisms Cosgrove et al., 2010.	No drug-induced inflammatory response
Ogese et al. (2017)	pHHs, drug-specific T cell, DCs	CM	24 h	SMX-NO	Flucloxacillin, INH, amoxicillin	Flucloxacillin and SMX-NO prime T cell activity (HMGB1).	/
Dragomir et al. (2011)	pMHs, RAW264.7	CM	24 h	NA	APAP	Upregulation of pro-inflammatory genes in RAW264.7 via HGMB1 travel	/
Kegel et al. (2015)	pHHs, pHKCs	CM	2 h	NA	APAP, DIC	Drug → cytokine Donor-matched	Donor-variable cytokine response
Goto et al. (2015)	pRKCs	CM	24 h	LPS (0.03)	APAP, TVX, TGZ	Drug → cytokine, IL-6/IL-1β ratio↓	No TVX-induced TNFα observed
Ogese et al. (2019)	pHHs, drug-specific T cell, DCs,	CM	24 h	SMX-NO	Flucloxacillin, INH, amoxicillin	Drug-specific hapten activates T cell via exosome	/
Kato and Uetrecht (2017)	FLC-4, THP1	CM	7 d	NA	Amodiaquine, Nevirapine	DAMPs released from FLC-4 activate inflammasome from THP1	/
Mak et al. (2018)	FLC-4, THP1, J774A.1	CM	7 d	NA	TGZ, tolcapone, entacapone	DAMPs released from FLC-4 activate inflammasome from THP1	/

*pHHs, primary human hepatocytes; pRHs, primary rat hepatocytes; pMHs, primary mouse hepatocytes; pRKCs, primary rat Kupffer cells; pKCs, primary human Kupffer cells; CM, conditioned media; SC, sandwich culture; DCs, dendritic cells; LPS, lipopolysaccharide; TNFα, tumor necrosis factor α; IFN, interferon; IL-1α, interleukine-1α; APAP, acetaminophen; TVX, trovafloxacin; DIC, diclofenac; TGZ, troglitazone; INH, isoniazid; SMX-NO, nitroso sulfamethoxazole; HGMB1, high-mobility group box 1 protein*.

Conversely, studies have also tested the effects of drug-exposed hepatocyte media (Dragomir et al., 2011; Kegel et al., 2015; Ogese et al., 2017) or direct addition of hepatotoxicants on immune cells including DCs/T cells (Ogese et al., 2017) and KCs (Goto et al., 2015) without including hepatocytes in the culture (Table 1). Adding hepatotoxicants and LPS to rat KCs caused an increase in pro-inflammatory cytokine interleukin-1 beta (IL-1β) and decrease in anti-inflammatory cytokines IL-6 and interleukin-10 (IL-10) (Goto et al., 2015). However, TNFα secretion remained unchanged which is contradictory to *in vivo* evidence (Shaw et al., 2010; Monga, 2011). An increase in reactive oxygen intermediates and changes in pro-and anti-inflammatory cytokines when KCs were exposed to APAP or Diclofenac (DIC)-treated hepatocyte conditioned media have also been reported (Kegel et al., 2015). Of note, this particular study highlighted the wide donor-dependent variability in cytokine responses across different batches of KCs. Exosomes of drug-treated hepatocytes have been shown to be phagocytosed by monocyte-derived DCs and T cells activated using drug-specific hapten (Ogese et al., 2019). Another study by the same group showed that conditioned medium from hepatocytes treated with im-DILI-associated drugs activated DCs through High-mobility group box 1 protein (HMGB1) and other DAMP molecules, which in turn stimulated T cells (Ogese et al., 2017). HMGB1 was also suggested in a different study to serve as a DAMP in acetaminophen-treated hepatocyte-conditioned media which activated macrophages to produce cytotoxic and proinflammatory mediators known to be involved in hepatotoxicity (Dragomir et al., 2011). In addition, the role of DAMPs in inflammasome activation was tested using human hepatocarcinoma liver cell line, FLC-4, treated with amodiaquine and nevirapine (Kato and Uetrecht, 2017). Drug-treated FLC-4 conditioned media activated THP-1 macrophages, and while amodiaquine could activate THP-1 directly due to possible metabolization by THP-1, nevirapine did not as it needs to be metabolized primarily by hepatocytes prior to inflammasome activation (Kato and Uetrecht, 2017). Similar inflammasome activation through DAMPs with tolcapone and troglitazone (TGZ)-treated hepatocyte conditioned media have also been reported (Mak et al., 2018). Overall, these studies suggest that hepatotoxicants themselves or DAMP molecules released by apoptotic or necrotic hepatocytes after initial drug injury can be a key triggering factor for the activation of immune cells in the liver. However, the presence of hepatocytes in culture is still required to elucidate their complex crosstalk with immune cells, necessitating the need for more complex co-culture studies.

### Co-cultures of Animal Cells

The majority of animal *in vitro* hepatocyte culture systems use pRHs, as they are easier to cryopreserve (Swales et al., 1996b) as well as maintain their metabolic functions better than mouse hepatocytes (pMHs) (Swales et al., 1996a). Recognizing the role of immune cells in the progression of DILI as seen in *in vivo* animal models, there has been some attempts to develop *in vitro* co-culture systems with both hepatocytes and immune cells. These approaches have mainly been restricted to monolayer cultures, although some inroads have also been made into 3 dimensional cultures containing other cell types such as stellate cells that better represent the *in vivo* environment (Kostadinova et al., 2013).

Given the abundance of macrophages within the liver, it is natural for the focus of immune-hepatocyte systems to be placed on KCs (Kurose et al., 1996; Milosevic et al., 1999; Tukov et al., 2006; Kostadinova et al., 2013; Bonzo et al., 2015; Rose et al., 2016) (Table 2). These cultures typically involve sorting the hepatocyte and macrophage populations from freshly isolated livers, before putting them back into the same culture. From these cultures, it is evident that some of the inter-cellular interactions are recapitulated *in vitro*, as stimulating these co-cultures often result in greater responses than that of either cell type alone. For example, rat KCs that have been pre-treated with LPS increased Nitrate/Nitrite content in the co-culture supernatant by 11-fold, as opposed to 7-fold in the case of KCs alone (Kurose et al., 1996). Interestingly, addition of anti-iNOS oligodeoxynucleotide (ODN) to the KCs led to an initial down regulation of Nitrate/Nitrite production in the co-culture after 2 h, which partially recovered at the experimental end-point of 4 h, while addition of the anti-iNOS ODN to the hepatocytes had the opposite effect: with normal Nitrate/Nitrite levels at the 2 h mark and attenuated expression at the 4 h time point. This suggested that KCs may have a priming and amplifying role in hepatocyte response to stress, in line with our understanding of them as integrative machines (Bleriot and Ginhoux, 2019).

**Table 2:** Animal *in vitro* liver co-culture models comprising of hepatocytes and immune cells.

**References**	**Cells**	**2D/3D**	**Duration**	**Stimuli (μg/ml)**	**im-DILI compounds**	**Findings**	**Disadvantages**
Kurose et al. (1996)	pRHs, LPS-pRKCs	2D	2/4 h	LPS (1)	/	LPS-pRKCs → TNFα and nitric oxide → Mt dysfunction in pRHs	No drug testing data
Milosevic et al. (1999)	pRHs, pRKCs (1)	Transwell	48 h	LPS (≤10)	/	LPS-induced cytokine in pRKCs reduced pRHs functions	No drug testing data
Rose et al. (2016)	pRHs, pRKCs (2.5)	2D	48 h	LPS (1)	APAP, TVX	↑ Cytotoxicity in co-cultures. Drugs altered levels of LPS-induced cytokines	No TVX-induced TNFα observed
Kostadinova et al. (2013)	pRHs, NPC (~1.5)	Transwell scaffold	15 d	LPS (10)	TGZ, TVX, APAP	↑sensitivity in co-cultures; species-specific effect observed.	Drug-induced inflammatory response not tested.
Tukov et al. (2006)	pRHs, pRKCs (0.25)	2D	6 h	LPS (0.0003 and 0.01)	APAP, chlorpromazine, monocrotaline	chlorpromazine, APAP → ↑TNFα monocrotaline → ↓ TNFα	
Bonzo et al. (2015)	pRHs, pRKCs (2)	2D	24/48 h	LPS (1)	TVX	TVX → TNFα ↑ Ratio of TNFα/IL-6 ↓ IL-6 downregulated CYP in pRHs	Only one drug tested
Goto et al. (2015)	pRKCs	2D	24 h	LPS (0.03)	APAP, TVX, TGZ	Drug → cytokine, IL-6/IL-1β ratio↓	No TVX-induced TNFα observed
Poulsen et al. (2014)	RAW264.7	2D	12 h (TVX-2, LPS-10)	LPS (0.01)	TVX <100 uM	TVX-induced TNFα (ERK, JNK)	Only one drug tested.

*Numbers in parenthesis in the “cells” column indicate ratio of hepatocytes: immune cells. Absence of numbers indicate that the study didn't specify the ratio of cell used. pRHs, primary rat hepatocytes; pRKCs, primary rat Kupffer cells; LPS, lipopolysaccharide; APAP, acetaminophen; TVX, trovafloxacin; TGZ, troglitazone; TNFα, tumor necrosis factor α; IL-6, interleukin-6; IL-1β, interleukin-1β; CYP, cytochrome P450*.

The use of these hepatocyte/KC co-culture systems also helped to validate the role of KCs in the LPS/TNFα induced TVX hepatotoxicity observed in the *in vivo* animal models. TVX toxicity in hepatocytes was much higher in the co-cultures and was exacerbated by the addition of LPS, while the addition of LPS and TVX to hepatocytes alone was much milder (Bonzo et al., 2015; Rose et al., 2016). However, pro-inflammatory cytokine, TNFα production did not increase in a dose-dependent manner upon LPS and TVX treatment as observed *in vivo* (Shaw et al., 2010). These observations are strongly indicative that although animal *in vitro* models are able to partially capture the cellular interactions involved in im-DILI, they are limited in recapitulating *in vivo* findings.

### Co-cultures of Human Cells

#### pHHs and pKCs Co-cultures

The vast majority of human *in vitro* co-cultures have focused on co-cultures involving KCs, which is not surprising since they are hepatic resident macrophages comprising of 80–90% of total macrophages in the human body (Ma et al., 2017). Primary Kupffer cells (pKCs) and pHHs [the gold standard cell source for hepatocytes (Zeilinger et al., 2016)] have been co-cultured in a variety of culture formats including 2D (Sunman et al., 2004), micropatterned (Zinchenko et al., 2006; McVay et al., 2013; Nguyen et al., 2015), microtissue (Nguyen et al., 2016; Jiang et al., 2019; Kermanizadeh et al., 2019a) and microfluidic cultures (Long et al., 2016; Chen et al., 2017; Sarkar et al., 2017) in order to include inflammatory components to *in vitro* liver models (Table 3, Figure 1). In some of these studies, the authors used a ratio of 10:1 pHHs:pKCs (Long et al., 2016; Chen et al., 2017; Sarkar et al., 2017) in order to match KCs content of about 10% in the liver (Jones and Summerfield, 1989). However, due to enrichment of KCs in the periportal region under inflammatory conditions *in vivo*, a ratio of 2.5:1 pHHs:pKCs (Jaeschke, 2007) might be a better representation of inflammatory conditions *in vitro* (Sunman et al., 2004; Nguyen et al., 2015). In majority of these studies, the co-cultures were stimulated with LPS or cytokines or a combination of both (Sunman et al., 2004; Nguyen et al., 2015; Long et al., 2016; Chen et al., 2017; Sarkar et al., 2017) with the aim of analyzing the inflammatory response mediated by pKCs and their effects on specific pHHs metabolism. However, the findings of the studies were focused on specific cytokine-hepatocyte interactions such as IL-2 or IL-6 mediated downregulation of CYP3A4 in pHHs (Sunman et al., 2004; Long et al., 2016), an improvement of hepatocyte function and drug metabolism as a result of the co-culture (Zinchenko et al., 2006; Sarkar et al., 2017) and better mimicry of *in vivo* interactions with other organs such as the gut (Chen et al., 2017). To the best of our knowledge, there are only two reports on the immune-mediated effects of a drug. One of them is a 3D InSight™ Human Liver Microtissue (InSphero) which was exposed to APAP (Jiang et al., 2019). APAP-induced production of interleukin-8 (IL-8) and IL-6 followed by a suppression of IL-6 in a dose dependent manner was reported. Transcriptomic analyses showed concurrent mitochondrial damage and oxidative stress responses. The results of this study must be interpreted carefully due to the high concentration of LPS used (10 μg/ml) (Jiang et al., 2019), which can cause significant toxicity even without the presence of the drug. The second one is a micropatterned triculture of pHHs, pKCs and fibroblasts (HepatoMune™, Hepregen/BioIVT) which demonstrated TVX-dependent TNFα increase at low concentrations followed by a decrease at higher concentrations (McVay et al., 2013), although consequent effects on pHHs were not tested. Unfortunately, only one drug was tested in both these models. The remaining culture systems mentioned above were not treated with drugs or compounds or even if they were treated with a single drug, immune-mediated effect of the drug was not tested. Hence the utility of these models in detecting immune-mediated DILI remains to be addressed.

**Table 3:** Human *in vitro* liver co-culture models comprising of hepatocytes and immune cells.

**References**	**Cells**	**Culture format**	**Duration of culture**	**Stimuli (μg/ml)**	**im-DILI compounds**	**Key findings**	**Disadvantages**
Sunman et al. (2004)	pHHs, pKCs (2.5/10)	2D	72 h	IL1 (0.0002-0.02) IL6 (0.002-0.2)	/	Proinflammatory cytokine inhibits CYP3A; pKCs required for IL-2 effect on CYP3A.	No drug testing data.
Zinchenko et al. (2006)	pHHs, pKCs (5/10)	MPCC/2D	10 d	/	/	Superior pHHs function in MPCC.	No drug testing data.
Nguyen et al. (2015)	pHHs+3T3, pKCs (2.5/10)	MPCC	<15 d	LPS (0.05) cytokines	/	Inflammatory stimulation systematically assessed	No drug testing data.
McVay et al. (2013)	pHHs, pKCs	MPCC	3/6 d	LPS (0.05)	TVX	TVX-induced TNFα (decrease > 200 μM)	Only one drug tested but using rat cells.
Kermanizadeh et al. (2019a)	pHHs, pKCs	InSphero InSight MT	7 d	/	nanoparticles	pKCs respond to pHHs damage	Donor variability; No drug testing data.
Jiang et al. (2019)	pHHs, pKCs (5)	InSphero InSight MT	24 h	LPS (10)	APAP (<10 mM)	↑ Cytotoxicity in co-cultures APAP enhanced LPS-induced IL-6 (<5 mM); reduced IL-6 by 50% at 10 mM	Only one drug tested.
Nguyen et al. (2016)	Liver tissue	Bioprinted	7 d	/	TVX	Long-term functions sustained; LPS-independent mechanism of TVX toxicity.	Drug-induced inflammatory response not tested; only one drug tested.
Chen et al. (2017)	pHHs, pKCs (10), intestinal cells	Microfluidic (LiverChip)	3/15 d	LPS (0.002)	/	Mimic gut-liver axis; Long-term functions sustained.	No drug testing data.
Long et al. (2016)	pHHs, pKCs (10)	Microfluidic (LiverChip)	<14 d	IL-6 (1)	Tocillzumab	IL6-CYP3A4 interaction tested.	No drug testing data.
Sarkar et al. (2017)	pHHs, pKCs (10)	Microfluidic (LiverChip)	48 h	LPS (1)	DIC (440 uM)	*In-vivo* like biotransformation of DIC; transcriptomics of co-culture	Drug-induced inflammatory response not tested; only one drug tested.
Messner et al. (2013)	pHHs, NPC	Spheroid	14d; <5 w	LPS (10)	APAP, TVX, DIC	↑ Cytotoxicity in the presence of LPS	Drug-induced inflammatory response not tested.
Kostadinova et al. (2013)	pHHs, NPC (~1.5)	Transwell scaffold	15d	LPS (10)	TGZ, TVX, APAP <10*Cmax	↑sensitivity in co-cultures; species-specific effect observed.	Drug-induced inflammatory response not tested.
Esch et al. (2015)	pHHs, NPC (~1.5)	Scaffold Kostadinova et al., 2013 microfluidic	14d	LPS (50)	/	pHH functions enhanced by flow; IL-8 production upon LPS.	No drug testing data.
Bell et al. (2016)	pHHs, NPC (2)	Spin spheroid	28 d	LPS (10)	DIC+4 more	↑sensitivity in co-cultures over time (w/o NPCs).	Drug testing results are based on pHH mono-culture.
Novik et al. (2010)	pHHs, NPC (1)	HμREL^®^ biochips	24 h	/	/	pHH functions enhanced by flow.	No drug testing data.
Granitzny et al. (2017)	HepG2 THP-1 (2.5)	Transwell	24/48 h	LPS (1), TNFα (0.1)	TVX, DIC, TGZ, KCL <20*Cmax	↑ Cytotoxicity in co-culture with THP-macrophage, but not with THP-monocyte	Drug-induced inflammatory response not tested.
Wewering et al. (2017)	HepG2, THP-1	Transwell	24 h	/	KCL	↑ inflammation in co-culture only	Only one drug tested.
Edling et al. (2009)	Huh7, THP-1 (2.5)	Transwell	24 h	/	TGZ	↑ Cytotoxicity and stress responses in co-culture	Drug-induced inflammatory response not tested; only one drug tested.
Fasbender et al. (2020)	Huh7, HepG2, pHHs; PBMCNK	2D	Drug-24 h, add NK	/	INH, KCL, promethazine, valproic acid	Upregulation of NK cell ligand on hep exposed to drugs; ↑ Cytotoxicity in co-culture	HLA-unmatched co-culture

*The models can be broadly categorized into three different groups: (1) co-cultures of pHHs with pKCs (2) co-cultures of pHHs with NPCs (non-parenchymal liver cells), and (3) co-cultures of hepatic cell lines (HepG2 and Huh7) and macrophage cell line THP-1. Numbers in parenthesis in the “cells” column indicate ratio of hepatocytes: immune cells. In some studies, two different ratios were used, indicated by two numbers in the parenthesis. Absence of numbers indicate that the study didn't specify the ratio of cell used. MPCC, micropatterned co-culture; MT, microtissue; LPS, lipopolysaccharide; IL-1, interleukin-1; IL-6, interleukin-6; TNFα, tumor necrosis factor α; APAP, acetaminophen; TVX, trovafloxacin; DIC, diclofenac; TGZ, troglitazone; INH, isoniazid; KCL, ketoconazole; NPCs, non-parenchymal cells*.

#### pHHs and Liver Non-parenchymal Cells (NPCs) Co-cultures

Studies using other sources of immune cells other than pKCs include pHHs co-cultures with NPCs (Novik et al., 2010; Kostadinova et al., 2013; Messner et al., 2013; Esch et al., 2015; Bell et al., 2016). NPCs offer the advantage of testing effects of other hepatic cells such as stellate cells and endothelial cells in addition to KCs. Disadvantages include limited availability, cost, donor variability (Kermanizadeh et al., 2019a) and difficulty in controlling the ratio of pHHs: each NPC cell type; for example, increasing KCs amount to mimic *in vivo* like inflammatory state would also increase the number of stellate cells, endothelial cells or fibroblasts in culture. Interestingly, a ratio of 1.5:1 pHHs: NPCs, was used for some of the studies (Kostadinova et al., 2013; Esch et al., 2015) with the intention to mimic 60% parenchymal and 40% non-parenchymal cells *in vivo*; however, this ratio doesn't mimic *in vivo* inflammatory state in terms of KCs where pHHs:KCs ratio is about 2.5:1 (Jaeschke, 2007), nor is it clear whether *in vivo* content of other NPCs such as stellate cells (5–8%) is being mimicked (Yin et al., 2013). Of note is also the concentration of LPS used in these studies: 10–50 μg/ml which is significantly higher than concentrations used in most co-culture studies (50 ng/ml to 1 μg/ml) (Nguyen et al., 2015; Granitzny et al., 2017; Sarkar et al., 2017; Tasnim et al., 2019) and physiologically relevant concentrations (Desch et al., 1989). Irrespective of the culture conditions, the studies showed that pHHs functions could be stabilized for long-term cultures (≥14 days) in the presence of NPCs, which was similar to findings in pHHs-pKCs co-cultures. Unlike pHHs-pKCs co-cultures, several drugs were tested in the pHHs-NPCs co-cultures: APAP (Kostadinova et al., 2013; Messner et al., 2013), TVX (Kostadinova et al., 2013; Messner et al., 2013), DIC (Messner et al., 2013; Bell et al., 2016) and TGZ (Kostadinova et al., 2013). In addition, Bell et al. treated their co-culture with Amiodarone, Bosentan, Fialuridine and Tolcapone (Bell et al., 2016). However, only the cytotoxic effects of the drugs were tested, showing improved sensitivity, i.e., detection of toxicity at lower concentrations in pHHs-NPCs co-cultures when compared to pHHs mono-cultures (Kostadinova et al., 2013; Messner et al., 2013; Bell et al., 2016). Immune-mediated effects of the drugs in the system were not tested.

#### Hepatic Cell Lines and THP-1 Co-cultures

Probably due to the cost and lack of availability of pKCs and NPCs, some studies have used alternative cell sources such as human hepatoblastoma cell line HepG2 and THP-1 monocytes/macrophages for the development of co-cultures (Granitzny et al., 2017; Wewering et al., 2017). The former reported increased cytotoxicity in THP-macrophage co-cultures, but not in THP-monocyte co-cultures in response to TGZ, TVX, DIC and Ketoconazole (KCL) in the presence of inflammatory stimulus: LPS and TNFα. The latter demonstrated co-culture specific induction of NRF2-mediated stress response and pro-inflammatory cytokines (CXCL8, TNFα and C-C motif ligand (CCL)-3) upon treatment with KCL. A co-culture of the human hepatoma cell line Huh7 and THP-1 showed that TGZ exposure led to an increase in gene expression of pro-inflammatory cytokines and stress-related genes in both cell types (Edling et al., 2009). Although pro-inflammatory cytokines such as IL-1β was elevated in the co-culture, TNFα, a key pro-inflammatory cytokine was in fact downregulated. This might be due to the fact that cell lines are often not functionally similar to primary cells as exemplified by the differences in cytokine profiles of THP-1, KCs and human blood-derived primary macrophages following compound exposure (Kermanizadeh et al., 2019b).

#### Hepatic Cell Lines and NK Co-cultures

Most studies involving hepatic interactions with immune cells other than macrophages (e.g., NK cells, DCs and T cells) have been conducted with drug exposed-hepatocyte conditioned media (section Soluble Factor-Driven Single Cultures in Combination With Inflammatory Mediators). There are limited reports of direct co-cultures of hepatocytes and these immune cells. The only report of a direct co-culture is a study of human hepatocyte cell lines Huh7/HepG2 and peripheral blood mononuclear cell (PBMC)-derived NK cells which demonstrated enhanced NK cell activation and upregulation of NK cell ligands in Huh7/HepG2 after exposure to isoniazid (INH), KCL, Promethazine and Valproic acid (Fasbender et al., 2020). Increased cytotoxicity and interferon (IFN)-γ production were observed. This study demonstrated that, in addition to macrophages, NK cells can modulate im-DILI through direct interactions with hepatocytes.

Despite these studies contributing to significant progress in the field, most published studies were unfortunately limited to a single or handful of drugs and exposure scenarios. Hence, further work needs to be done for the establishment of a general im-DILI testing system that can be applied to structurally and mechanistically diverse im-DILI-associated compounds. One major challenge in performing such comprehensive studies is the lack of a source of relevant, functional cells that can be generated in large numbers. Cell lines (macrophages: mouse RAW264.7, human THP cell lines and hepatocytes: HepG2 and Huh7) can resolve the cell number issue but often do not represent the complex functions of primary cells (Kaur and Dufour, 2012). On the other hand, primary cells are expensive, show donor variability (Stoddart et al., 2012; Kegel et al., 2015; Kermanizadeh et al., 2019a), not easily accessible and cannot be expanded extensively in *in vitro* culture (Honegger, 1999).

#### Induced Pluripotent Stem Cells Co-cultures

Generation of induced pluripotent stem cells (iPSCs)-derived cell types provide an attractive alternative due to their accessibility, renewability and scalability (Shi et al., 2017; Doss and Sachinidis, 2019). Although generation of iPSC-derived hepatocytes (iHeps) have been established over the past decade both by us (Tasnim et al., 2015, 2016; Mittal et al., 2016) and others (Hannoun et al., 2016; Maepa and Ndlovu, 2020), iPSC-derived macrophages (iMacs), especially iPSC-derived KCs (iKCs) and other immune cells generated from iPSCs (Trump et al., 2019) are still in their infancy (Lee et al., 2018). We have recently published the first report to generate iKCs from iPSC-derived macrophage precursors (preMϕ) by providing the preMϕ with hepatic cues (Tasnim et al., 2019). The transcriptomic profile of iKCs was distinct from bone marrow-derived macrophages, alveolar macrophages, non-liver macrophages as demonstrated by principal component analysis. iKCs were similar to pKCs in terms of marker expression and functions and could mimic pKCs in detecting hepatotoxicity of im-DILI associated drugs APAP and TVX when co-cultured with pHHs or iHeps (Tasnim et al., 2019). This is not trivial since iPSC-derived cells are often reported to be immature and have lower functions compared to their primary counterparts (Doss and Sachinidis, 2019; Volpato and Webber, 2020). We are currently working on two different aspects to improve and further validate our co-culture model.

The first aspect is a direct co-culture of iHeps and iMacs for providing iMacs with a hepatic niche in order to differentiate toward a hepatic linage i.e., iKCs (in our previous study iKCs were generated using hepatic conditioned media and not a direct co-culture with hepatocytes (Tasnim et al., 2019). Other than providing direct cellular interactions, this system has the advantage of allowing generation of iKCs and iHeps from the same donor. Hepatocytes cultured with KCs (Tasnim et al., 2019) and T cells (Figueiredo et al., 2019) from different donors have shown activation of the respective immune cells. Alloreactivity could be reduced by silencing of HLA I on pHHs (Figueiredo et al., 2019). Donor-matched iKCs and iHeps developed by us would not require such additional measures to suppress immune reactions.

The second aspect we focus on is the application of the co-culture model to a diverse array and larger number of im-DILI associated drugs to validate if our model can stratify drugs based on their immune-mediated effects. Detailed cytokine production by iKCs upon treatment with each drug and their consequent effect on iHeps are being assayed. We are also analyzing correlation of the *in vitro* findings to clinical data to assess the physiological relevance of our model. Finally, such model might provide hints toward more sensitive hepatotoxicity endpoints as opposed to conventional cell viability assays.

## Technical Challenges in Establishing Co-Culture

Despite the benefits of incorporating immune cells to investigate im-DILI, some key technical questions need to be addressed for co-culturing hepatocytes and immune cells: (1) How do we obtain a steady source of the appropriate immune cells, especially for human cultures? (2) For how long do functional co-cultures need to be maintained? (3) How do we balance the use of components essential for one cell type but harmful for the counterpart, such as serum and growth factor? (4) Are immune cells primed when being in contact to HLA-unmatched hepatocytes? (5) How well do *in vitro* cells recapitulate human pathophysiology?

Although similar difficulties also exist when trying to model hepatocyte only cultures, obtaining the appropriate human resident immune cells, KCs in this case, poses a unique challenge. Even though KCs are the largest immune population in the liver, they still only make up 10% of the liver, which makes obtaining and using primary KCs in co-culture screening models prohibitively expensive. Blood monocytes have been explored as a possible surrogate for KCs in co-culture models (Rennert et al., 2016), however with our current understanding of macrophage ontogeny (Ginhoux and Jung, 2014; Perdiguero et al., 2015), they might be an inadequate substitute. We now know that, as seen in the various murine fate mapping models (Hoeffel et al., 2015), human KCs are also initially seeded by non-hematopoietic stem cell derived embryonic macrophages (Bian et al., 2020). This has far-reaching consequences, as embryonic macrophages have been shown to maintain a distinct transcriptomic and epigenetic landscape in the murine models and are self-renewing well into adulthood under steady state conditions (Lavin et al., 2014). Thankfully, this is not an insurmountable challenge, as murine iMacs have been shown to be similar to embryonic yolk sac macrophages (Takata et al., 2017), and so human iMacs, especially in a hepatic environment, may prove to be a suitable KC surrogate in im-DILI co-culture models.

Developing a suitable KC surrogate is also dependent on overcoming a separate but related problem: defining human KC identity and their development and specification trajectories. Although the differentiation of murine KCs is well-studied (van de Laar et al., 2016), some of the markers used to distinguish KCs from macrophages are not applicable to humans. An example of this would be CLEC4F, which is considered a definitive KC marker in mice, but does not encode for a functional receptor in humans (Taylor et al., 2019). Furthermore, using a handful of surface markers to claim identity of cells raised *in vitro* can be misleading, as cells can downregulate or even lose expression of markers after prolong culture. This limitation is starting to be addressed by the increased adoption of high dimensional techniques such as bulk and single cell RNAseq (scRNAseq) and ATACseq, allowing for a more holistic view of macrophage development and tissue interactions. For instance, scRNAseq has allowed us to observe the step-wise transcriptomic changes that occur along KC specification in the human embryo (Bian et al., 2020), providing a reference framework for *in vitro* KC development. The large volume of data generated by these techniques also enables the use of powerful new tools such as CellPhoneDB to interrogate macrophage-tissue interactions (Efremova et al., 2020), which can help us to better understand injury mechanisms and identity therapeutic targets in future im-DILI models.

Long-term cultures are important for recapitulating the latency of im-DILI (Chalasani et al., 2015), which can be intermediate (1–8 weeks) or long (up to 12 months) (Liu and Kaplowitz, 2002). It is challenging to maintain the morphology and functions of hepatocytes on common 2D culture over extended periods (Richert et al., 2006). Long-term hepatocyte cultures have been maintained using more complex designs including 3D cultures (Mittal et al., 2016; Tasnim et al., 2016; Lauschke et al., 2019) and co-cultures [mainly with fibroblasts (March et al., 2015)]. Some of the long-term culture models which involve co-culture with immune cells have been described in section Co-cultures of Human Cells and Table 3. Hepatocyte functions were well-maintained for at least 1–2 weeks in both serum-containing (Zinchenko et al., 2006; Kostadinova et al., 2013) and serum-free conditions (Messner et al., 2013; Nguyen et al., 2015, 2016; Bell et al., 2016; Long et al., 2016) with the aid of supportive mechanical environment such as microtissue, microfluidics and spheroids. However, complex culture systems are not conducive to high throughput applications. When a less complex high-throughput model is required for long-term culture, optimization of medium conditions becomes necessary.

Medium is a critical factor for optimal cell function *in vitro*. This comprises of the basal medium and supplements including serum. Various immune cells share similar culture conditions: RPMI 1640 (basal medium) with 10% serum, sometimes supplemented with growth factors, is commonly used (Moore et al., 1967; Exley et al., 2010). Use of RPMI 1640 for hepatocyte culture has also been reported (Burdon and van Knippenberg, 1991) and was comparable to William's E Medium (WEM) up to 6 days of culture (Nelson et al., 2013). Hence, RPMI 1640 (without serum) could be used for short-term co-culture of hepatocytes and immune cells. In agreement with this, Sana et al. for instance, used RPMI 1640 for co-culture of hepatocytes and PBMCs and DCs for up to 96 h (Sana et al., 2014). The effect of RPMI 1640 or other suitable basal media (e.g., WEM) on long-term co-cultures of hepatocytes and immune cells needs to be addressed.

Compared to basal medium, the use of serum is more contradictory. On one hand, despite the heterogeneity of serum composition from different lots and origins, it is essential for *in vitro* expansion and for obtaining optimal cell functions of immune cells (Musson, 1983; Costello et al., 2000). On the other hand, serum is deleterious to human hepatocyte culture due to inhibition of important cell functions (Enat et al., 1984). In most cases, hepatocytes favor serum-free medium supplemented with insulin-transferrin-selenium (ITS) and dexamethasone (Chard et al., 1987). We postulate that serum-free medium should be developed for co-cultures of hepatocytes and immune cells. A serum-free condition has been established for human KC culture by Tasnim et al. (2019). KCs were cultured with advanced DMEM plus Cocktail B^TM^ (containing ITS) and in WEM plus Cocktail B^TM^ when co-cultured with hepatocytes without functional compromise (Tasnim et al., 2019). Serum-free medium for immune cell cultures has been being development for decades, including AIM V^TM^ and X-VIVO15^TM^ (Medvec et al., 2018), but has yet to be tested for hepatocytes' functions, especially for long term co-cultures.

Another important challenge which might often go unnoticed is the frequency of media change. On one hand, a high frequency of media change is not conducive to the interaction of hepatocytes and immune cells through soluble factors. On the other hand, hepatocytes are highly metabolic cells and hence require frequent media change (typically, hepatocyte media is changed daily). Therefore, co-culture conditions need to be tuned carefully in order to strike a delicate balance. To this effect, we have optimized a method to co-culture iMacs and iHeps for up to 1 week with ideal media change frequency and re-defined media supplement concentrations (unpublished data). It will be interesting to assess if these conditions work for longer time periods and other immune cell co-cultures.

Notably, glucocorticoids (GCs), such as dexamethasone, is essential to maintain hepatocytes' functions particularly in extended period (Bailly-Maitre et al., 2001); however, they pose potent anti-inflammatory effects on immune cells, including macrophages (Werb, 1978; Rose et al., 2016), NK cells (Bush et al., 2012) and T cells (Ashwell et al., 2000). We also observed significant inhibitory effects of dexamethasone on pKCs and iKCs (data not shown). Conversely, low concentration of corticosterone (0.1 nM) was reported to enhance pro-inflammatory responses in rat PBMC (Lim et al., 2007). Despite such discrepancy, there are two options to resolve this: (1) Co-culture without dexamethasone which did not show any functional compromise in iHeps for short-term cultures (Tasnim et al., 2019) or (2) Selection of an appropriate GC to both maintain hepatocyte functions and retain cytokine production from KCs as demonstrated by Rose et al. (2016). Their results suggested that a less potent GC hydrocortisone might be used during the plating phase for the co-culture. However, whether these two options are applicable for long-term cultures needs to be systematically examined.

In addition, the need for HLA-matched cells in co-cultures should also be addressed. Due to limited accessibility to hepatocytes/immune cells, it is challenging to frequently obtain cells from the same donor. Many of the co-culture studies have to proceed with cells from different donors, indicating extremely low possibility of obtaining HLA-matched co-cultures. This might pose a challenge as HLA-unmatched co-cultures have shown to activate KCs (Long et al., 2016; Tasnim et al., 2019) and T cells (Figueiredo et al., 2019). Even though co-culture with transwell setup could attenuate the pre-activity of immune cells by reducing physical contact, it was also reported that HLA could travel through exosome to stimulate other immune cells (De Toro et al., 2015) or the paracrine factors secreted from immune cells could be sufficient for alloreactivity (Figueiredo et al., 2019; Tasnim et al., 2019). Strategies to reduce such activity include the use of the immune cells harboring the most frequent HLA class, or leveraging on donor-matched cells derived from human iPSCs.

Finally, careful consideration must be given to the interpretation of any functional readouts obtained from the stimulation of *in vitro* cells. These co-culture model systems are useful for the understanding of cell-to-cell interactions under specific defined conditions, and we should be careful not to overinterpret them in relation to complex human pathophysiology. As seen in a few of the studies mentioned previously, non-physiological concentrations of stimulants are sometimes used, partially in recognition of the decreased metabolic sensitivity of cells in culture, but often simply to obtain a response from these cells. One way around this issue might be the integration of *in silico* approaches with *in vitro* experiments. Using meta-analyses, possible pathways of im-DILI can be identified (Selvaraj et al., 2020), then experimentally tested using the relevant human *in vitro* systems. The results from these experiments can then be compared to clinical diagnoses and observations from human patients, giving a more holistic view of the adverse event.

## Conclusion and Future Perspectives

Immune-mediated toxicity can involve many different cell types in various locations and can develop over a wide timeframe. A conceivable mechanism of im-DILI that has been proposed starts from reactive metabolite formation in the liver, followed by drug-modified proteins and release of DAMPs (Uetrecht, 2019). This in turn involves migration of antigen presenting cells to lymph nodes and spleen, where T cells are activated and return to the liver to mediate injury. It would be extremely challenging to duplicate this process *in vitro*; not only due to the complexity of the culture system involving hepatocytes, KCs, DCs and T cells, but also because the system has to be architecturally designed to mimic the sequence of events. Therefore, caution needs to be exercised when extrapolating the data obtained from *in vitro* studies to human pathophysiology. Specifically, it would not be appropriate to deduce immune-mediated injury mechanisms from the results of these studies unless predictions obtained from the data are consistent with characteristics of immune-mediated toxicity in patients. In light of this, our recent work has focused on identifying drugs that have clear immune-mediated responses in patients (e.g., changes in specific cytokine levels upon drug treatment) and validating if those specific changes are recapitulated in our *in vitro* model (unpublished data). In this manner, we could recapitulate certain elements of immune-mediated toxicity *in vitro*. Nevertheless, further efforts need to focus on improving the performance of *in vitro* models so that they can reproduce more aspects of human pathophysiology. In this regard, we postulate several strategies. Systematic evaluation of conditions for long-term co-cultures and development of microfluidic chip for temporal control of intercellular interaction are encouraged. While classical studies with primary cells and cell lines have helped to advance the field immensely; we now have more options for cell sources through establishment of iPSC-derived immune cells. Hence, renewable and functional cell source of other immune cells could be developed from human iPSC to overcome the low accessibility of liver cells and to facilitate establishment of HLA-matched co-cultures. Through this review, we highlight the gaps in investigating im-DILI *in-vitro* in order to encourage multi-disciplinary efforts to improve the understanding and modeling of im-DILI. Such developments will greatly enhance drug safety assessment, especially in terms of predicting immune reactions elicited by drugs.

## Author Contributions

All authors listed have made a substantial, direct and intellectual contribution to the work, and approved it for publication.

## Conflict of Interest

The authors declare that the research was conducted in the absence of any commercial or financial relationships that could be construed as a potential conflict of interest.
